# Potential Genes and Pathways of Neonatal Sepsis Based on Functional Gene Set Enrichment Analyses

**DOI:** 10.1155/2018/6708520

**Published:** 2018-07-30

**Authors:** YuXiu Meng, Xue Hong Cai, LiPei Wang

**Affiliations:** ^1^Department of Neonatology, First People's Hospital of Jining, Jining, Shandong 272000, China; ^2^Department of Pediatrics, Traditional Chinese Medicine Hospital of Yanzhou, Jining, Shandong 272100, China

## Abstract

**Background:**

Neonatal sepsis (NS) is considered as the most common cause of neonatal deaths that newborns suffer from. Although numerous studies focus on gene biomarkers of NS, the predictive value of the gene biomarkers is low. NS pathogenesis is still needed to be investigated.

**Methods:**

After data preprocessing, we used KEGG enrichment method to identify the differentially expressed pathways between NS and normal controls. Then, functional principal component analysis (FPCA) was adopted to calculate gene values in NS. In order to further study the key signaling pathway of the NS, elastic-net regression model, Mann–Whitney *U* test, and coexpression network were used to estimate the weights of signaling pathway and hub genes.

**Results:**

A total of 115 different pathways between NS and controls were first identified. FPCA made full use of time-series gene expression information and estimated *F* values of genes in the different pathways. The top 1000 genes were considered as the different genes and were further analyzed by elastic-net regression and MWU test. There were 7 key signaling pathways between the NS and controls, according to different sources. Among those genes involved in key pathways, 7 hub genes, PIK3CA, TGFBR2, CDKN1B, KRAS, E2F3, TRAF6, and CHUK, were determined based on the coexpression network. Most of them were cancer-related genes. PIK3CA was considered as the common marker, which is highly expressed in the lymphocyte group. Little was known about the correlation of PIK3CA with NS, which gives us a new enlightenment for NS study.

**Conclusion:**

This research might provide the perspective information to explore the potential novel genes and pathways as NS therapy targets.

## 1. Introduction

Neonatal sepsis is the most prevalent cause of death of the neonates with few certainly reported biomarkers for many years. At least 35% neonatal deaths were caused by infections each year. The neonates have usually suffered from early-onset NS, which occurs within the first 72 hours after birth [1, 2]. According to the recent report, the diagnosis of sepsis is humbled by the nonspecific and highly variable human inflammatory and anti-inflammatory processes [3]. The main risk factor that causes the neonatal death is infections, which include respiratory infections, drug-resistant infections, and neonatal tetanus [4]. In order to manage the infections, the research to develop primary and secondary prevention strategies based on different kinds of infections has been a hot field for NS study in recent decades [5, 6]. Future medical research should be based on reducing the application and duration of antibiotics for NS. In view of the side effects caused by the treatment of NS, it is important to make the division for using the right standard of practice for the vulnerable group. The current classification criteria for susceptible populations are crucial to future research and to improve the development of neonatal management strategies. Medzhitov et al. found that the Toll-like receptor-2 and Toll-like receptor-4 are involved in the recognition process of the bacteria in neonates [7]. Septic neonates have a significant upregulation and obvious decline of several genes, which involved in innate immunity [8, 9]. The neonatal innate immune response to sepsis is driven by innate immunity genes (IL1R2, ILRN, and SOCS3) [10]. Current studies also investigated the relationship between the cytokine pattern and onset of NS and proved that the increased expression of proinflammatory cytokines, such as TNF-alpha, IL-6, and IL-10, was associated with the acute and post-acute phase of NS, respectively [11].

Despite the numerous studies of NS pathogenesis based on genes, the valuable predictors have remained unveiled and contributed to being a major challenge to the research of NS. Gene transcriptomic profiles can be used to identify diagnostic and prognostic gene signatures in complex diseases and to reveal the pathogenesis of NS [9, 10]. Several systems biology approaches were built to dissect the physiological mechanism of sepsis. In particular, methods for discovering the context-specific activations of pathways [12, 13] were merged. However, for the time-course studies, it will be difficult to do clinical trials if the role of the gene of choice is not specific to the biological process of interest. In other words, a temporally differentially expressed gene should show a significant nonconstant expression pattern across time points. To address this, weighting methods were needed to be carried out to assess the functional similarities between a given gene and the sets in different time points [14].

In this report, a method based on the functional principal component analysis (FPCA) was proposed to discover arbitrary nonconstant trends in time-course data analysis [15]. After estimating the impact of the gene, an elastic-net regression model was used to analyze the weights of genes. Besides, a generalized Mann–Whitney *U* (MWU) test was also applied for gene set-level inferences. Finally, hub genes were determined by the topological feature of coexpression networks [16]. Using the proposed analysis method, susceptible pathways and crucial genes will be revealed. And they will facilitate the future investigation of NS.

## 2. Methods

### 2.1. Data Recruitment and Preprocess

Gene expression profiles of human peripheral blood cells at various time points from samples of meningococcal sepsis were deposited at Gene Expression Omnibus database with the data accession no. GSE11755, including NS patients and normal controls. These datasets were processed on Affymetrix Human Genome U133 Plus 2.0 Array platform. Totally, forty-one samples, which were drawn at four time points (*t* = 0, *t* = 8, *t* = 24, and *t* = 72 h after admission to the paediatric intensive care unit), were studied, and key pathways and hub genes were also identified. Next, based on the RNA microarray, gene expressions isolated from whole blood, lymphocytes, and monocytes were also analyzed, respectively. According to the different sources of microarray data, we adopted different groups: The first, we named All Sources, contained all the 41 samples (10 controls and 31 patients). The second, we named Blood Source, contained the microarray data derived from blood (3 controls and 8 patients). The third, we named Lymphocyte Source, contained the microarray data derived from lymphocytes (4 controls and 12 patients). The fourth, we named Monocyte Source, contained the microarray data derived from monocytes (3 controls and 11 patients). In the following analyses, we conducted four parallel analyses based on different groups. The study was approved by the local medical ethics committee.

For data preprocessing, a freely available R platform (http://cran.r-project.org/) was applied. GraphPad Prism 7.0 software was used to create images. And data preprocess of dataset was commenced with reading the data by the standard method carried out by Affy. Expressions of genes were normalized using the robust multiarray average (RMA) method, in order to eliminate the influence of nonspecific hybridization [17]. And then, genes were further filtered by quartile-based algorithm [18]. A total of 15144 genes were reported for each subject.

### 2.2. Pathway Enrichment Analysis

Pathway analysis was used to find the significant pathways of the NS and control groups according to Kyoto Encyclopedia of Genes and Genomes (KEGG) [19]. Fisher's exact test was adopted to select the significant pathways, and the threshold of significance was defined by FDR and *p* value. Significant pathways were extracted according to the thresholds of *p* < 0.05 and intersection gene count >1.

### 2.3. A Gene-Level Summary Statistic by the Functional Principal Component Analysis

In the present research, the FPCA model was used to identify temporally differentially expressed genes [20]. The gene expression profile obtained was assumed to be the scattered members from the true profile of gene expression. And the true profile will be further interfered by noisy signals. After subtracting the average expression value of genes, FPCA was used to center all the gene values. The gene expression profile of preprocessed data was weighted according to their corresponding mean expression and FPCA score across all the gene expression values.

The observed expression using the FPCA model is as follows:

(1) $$\begin{array}{c} {\hat{X}}_{i}\left(t\right)={\hat{\mu }}_{i}+\overset{L}{\underset{l=1}{\sum }}{\hat{\xi }}_{il}{\hat{\Phi }}_{l}\left(t\right), \end{array}$$

where ${\hat{\mu }}_{i}$ is the average expression of the temporal sample, ${\hat{\Phi }}_{l}\left(t\right)$ is the *l*th eigenfunction, and ${\hat{\xi }}_{il}$ is the FPC value that quantifies how much ${\hat{X}}_{i}\left(t\right)$ can be explained by ${\hat{\Phi }}_{l}\left(t\right)$.

When it applied to the time-course gene expression, we used functional *F*-statistic to summarize the gene pattern information for each gene in the time points:

(2) $$\begin{array}{c} F_{i}=\frac{\mathrm{RS}S_{i}^{0}-\mathrm{RS}S_{i}^{1}}{\mathrm{RS}S_{i}^{1}}, \end{array}$$

where RSS_*i*_^0^ is the residual sum of squares of null hypotheses and RSS_*i*_^1^ is the residual sum of squares of alternative hypotheses. *F*_*i*_ can be viewed as a “signal-to-noise” ratio and revealed the importance of genes.

### 2.4. Estimating the Weights of Signaling Pathway Using the Elastic-Net Regression Model

In this study, we also took an approach with computationally efficient and highly flexible methods on the basis of an equivalent influence between the penalty function regression and a standard multivariate regression, in order to minimize optimization problem, which is known as the functional elastic-net regression problem [14]. This problem occurs because of the model selection methods in a functional linear regression model that is needless for the concurrent function regression.

The main function of the model is as follows:

(3) $$\begin{array}{c} \hat{\beta_{i}}=\underset{\beta_{i}}{\min}\mathrm{OBJ}\left(\beta_{i}\left|x_{i}\left(t\right)\right.,{\hat{\Phi }}_{i}\left(t\right)\right)\\ \;\;\;\mathrm{OBJ}\left(\beta_{i}\left|x_{i}\left(t\right)\right.,{\hat{\Phi }}_{i}\left(t\right)\right)={\left\|x_{i}\left(t\right)-{\hat{\Phi }}_{i}{\left(t\right)}^{T}\beta_{i}\right\|}^{2}+\lambda_{1}{\left\|\beta_{i}\right\|}_{1}+\lambda_{2}{\left\|\beta_{i}\right\|}^{2}, \end{array}$$

where *λ* is the penalty coefficient and *β*_*i*_ is the vector of the set of linear coefficients. When ${\hat{\beta }}_{i}$ is calculated and estimated, then the weights of the pathways can be obtained by

(4) $$\begin{array}{c} {\hat{W}}_{i,k}≔\frac{\sum_{l=1}^{L}{\left({\hat{\beta }}_{l,i}^{k}\right)}^{2}}{\sum_{k\in K_{i}}\sum_{l=1}^{L}{\left({\hat{\beta }}_{l,i}^{k}\right)}^{2}}. \end{array}$$

A similar approach can be used to estimate the weights of genes.

### 2.5. Weighted Mann–Whitney *U* (MWU) Test with Correlation Using Gene Set Enrichment Analysis (GSEA)

MWU test is used to compare two independent samples. Given that two samples were exactly from the same groups, the mean was different. The aim of the MWU test was to analyze whether there was a significant difference between the means of the two groups. Recent reports showed that MWU test plays an important role in gene set enrichment analysis (GSEA) [21, 22]. The pathway enrichment analysis was carried out based on the genome-wide background and was applied to identify the biological functions of the significant clusters. KEGG pathway enrichment was also performed. Categories with more than 5 genes were presented, and *p* value < 0.01 were considered significant in pathway enrichment analysis [23].

### 2.6. Identification of Hub Genes Based on the Coexpression Networks

Adjacency matrixes were firstly constructed based on the intergenomic relationships evaluated by Spearman correlation coefficient [24]. Topological features were further studied to find key nodes in the network. Genes whose degree was greater than the average degree values and whose Spearman correlation coefficient was greater than 0.6 were considered as hub genes.

## 3. Results

### 3.1. Pathway Enrichment Analysis

Gene expression profile of human NS with the series of GSE11755 was downloaded from Gene Expression Omnibus. After preprocessing the expression profile data of the dataset, we collected data from a total of 41 samples, including six children with meningococcal sepsis. Blood was drawn at four time points and matched with controls. Pathway enrichment analysis of NS and controls was conducted on the basis of the KEGG pathway database. A total of 286 pathways covering 6893 genes were obtained. After Fisher's exact test, 115 differential pathways covering 3532 genes met the thresholds of *p* < 0.05 and intersection gene count >1.  Table 1 shows the top 6 differential signaling pathways in ascending order based on *p* value.

### 3.2. Integrated Analysis of Gene Signatures Using the FPCA Model

**Figure 1:**
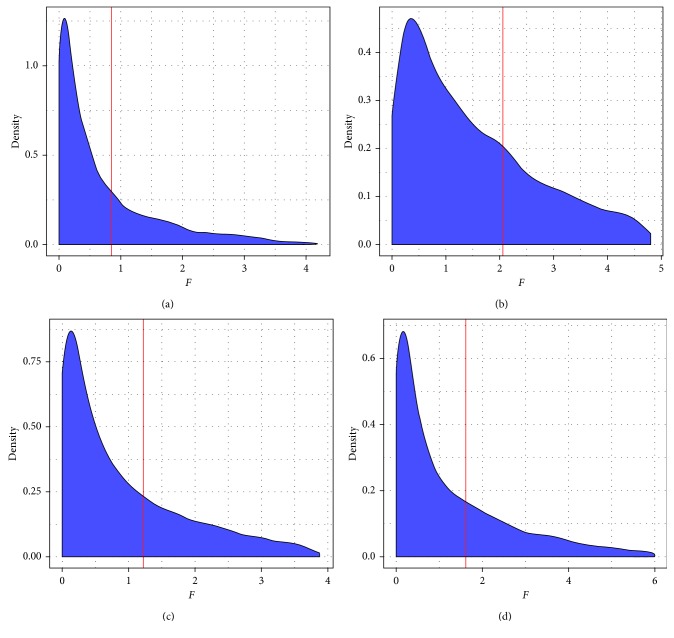
The distribution of *F* value of pathway genes. Time-series gene signatures data were analyzed by FPCA and each gene obtained an *F* value (*x*-coordinate, *F* value). *Y*-axis represents gene density. The genes were ranked in the order of *F* value, and the top 1000 of them were selected. The red line represents the threshold of top 1000 genes. (a) All Sources, (b) Blood Source, (c) Lymphocyte Source, and (d) Monocyte Source.

In the present research, the FPCA model was used to identify temporally differentially expressed genes and each gene would get an *F* value. Based on the 115 differential pathways (covering 3532 genes), we identified top 1000 gene signatures of NS using FPCA model, which were defined as dysregulated genes. FPCA narrowed the gene search range from 3532 to 1000. Greater *F* value means that the expression level differed greatly with others. Figures 1(a)–1(d) show the curve of gene signatures with *F* value. Among the dysregulated genes, the top 12 genes from All Sources, Blood Source, Lymphocyte Source, and Monocyte Source were CDC37, NCOA2, P2RY12, RXRB, EDEM2, ACTN4, STX12, PPM1A, PRKACB, DUSP10, VEGFA, and SLC44A2. Since NS is mainly caused by infections, the dysregulated genes in NS should be immune response related. However, there were few genes in the list that were immune related. Activation of the cytokines in a specific infection might not be derived from all the regulated genes that can activate those genes. Therefore, it is important to find the pathways which are activated by infections in NS. FPCA could effectively utilize the time-series information and overcome the traditional control design deficiencies [14]. *F* values would be used for the MWU test.

### 3.3. Estimating the Weights of Genes Using the Elastic-Net Regression Model

**Figure 2:**
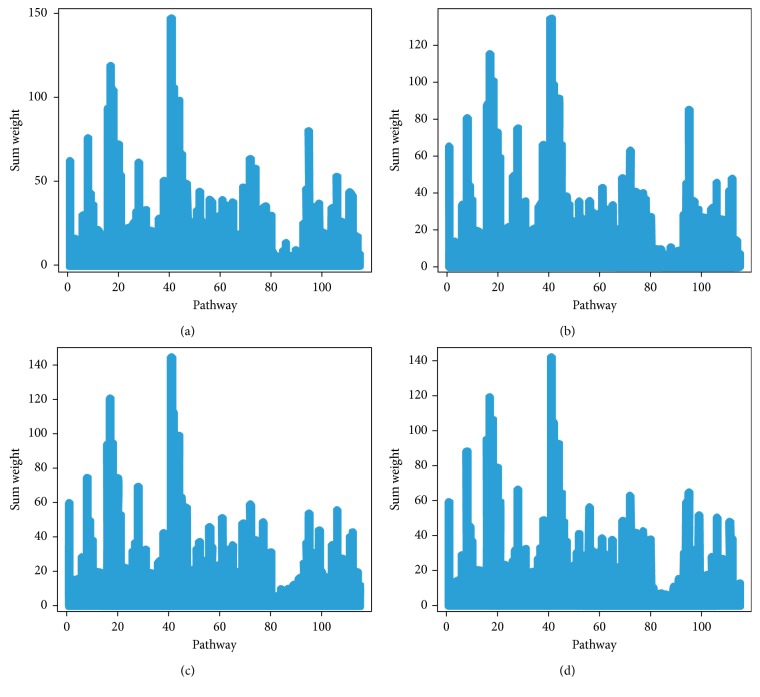
Sum weights of 115 differential pathways. *Y*-axis represents the sum weights of pathways. *X*-axis represents the number of pathways. (a) All Sources, (b) Blood Source, (c) Lymphocyte Source, and (d) Monocyte Source.

Genes that exist in multiple pathways were considered as overlapping genes. These genes are thought to play multiple roles in hypothesis testing, where the weight coefficients were overestimated. In the present study, elastic-net regression model was used to decompose an overlapping gene between gene sets and eliminate the overlapping effects. After calculating the weight value of each gene and adding the weight values of the pathway genes, the total weight value of the pathways was obtained.  Figure 2 shows the sum weight of each pathway. The weight value (*w*) of each gene would be used for the MWU test.

### 3.4. Functional Enrichment Analysis Using GSEA and MWU Model Test

Based on the KEGG pathway enrichment, 115 differential pathways were obtained. In order to more accurately find key pathways and molecules, FPCA and elastic-net regression were performed to eliminate overlapping gene effects. Combined with the MWU test, key molecular pathways in the gene transcription data of NS and controls were identified. Based on the *t*-test, pathways were ranked in the descending order. After the pathway data were tested by the MWU model, a total of 7 pathway terms met the condition *p* values < 0.05. There was no pathway met the conditions in the monocyte group. The resulting pathways are presented in Table 2.

**Figure 3:**
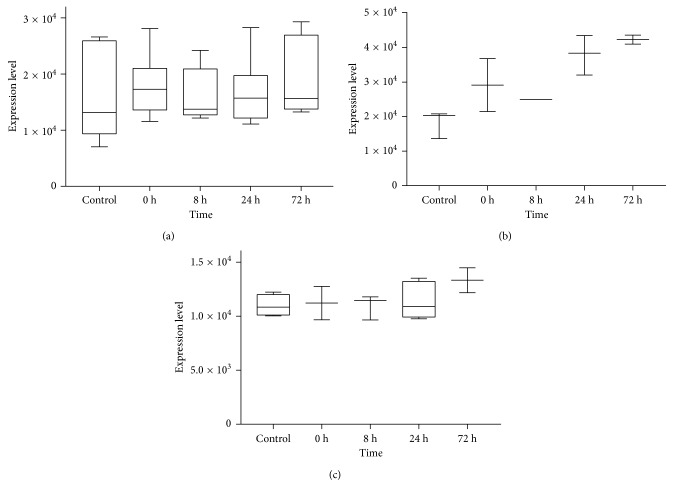
Expression levels of the top 3 significant signaling pathways. (a) hsa05220: Chronic myeloid leukemia from All Sources, (b) hsa05120: Epithelial cell signaling in *Helicobacter pylori* infection from Blood Source, and (c) hsa05222: Small-cell lung cancer from Lymphocyte Source. *Y*-axis represents expression levels of pathways. *X*-axis represents control and several time points after admission to the paediatric intensive care unit. The graphs were made with GraphPad Prism 7.0.

According to the MWU test, there were 7 pathways were screened based on the *p* values < 0.05. We selected the top 3 significant pathways: hsa05220: Chronic myeloid leukemia; hsa04380: Osteoclast differentiation; and hsa05222: Small-cell lung cancer for further analysis. Besides, pathways including the proinflammatory cytokine genes were also studied, such as hsa05164: Influenza A (TNF, IL-6, IL-18, and IFNA1; *p* value 0.3256); hsa04620: Toll-like receptor signaling pathway (TNF, IL-6, and IFNA1; *p* value 0.2185); hsa05168: Herpes simplex infection (TNF, IL-6, IFNA1, and IL-15; *p* value 0.4868). Unfortunately, the MWU test showed that there was no difference between the controls and patients in those proinflammatory cytokines included pathways. For the obtained genes in the top 3 pathways, Figure 3(a) reveals that the expression change of hsa05220: Chronic myeloid leukemia from All Sources was not obvious. The levels of hsa05120: Epithelial cell signaling in *Helicobacter pylori* infection from Blood Source after admission to the paediatric intensive care unit were significantly higher than control. Besides, the levels of hsa05222: Small-cell lung cancer from Lymphocyte Source were up at 72 h after admission to the paediatric intensive care unit.

### 3.5. Identification and Estimation of the Weights of the Hub Genes in the Pathway

**Figure 4:**
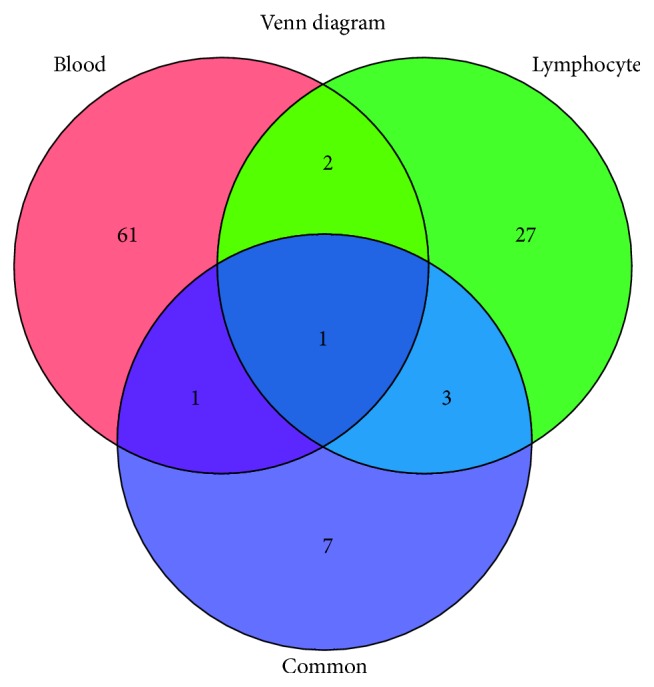
Venn diagram of hub genes based on the coexpression networks. Venn diagram showing the number of hub genes obtained from All Sources (Blue), Blood Source (Red), and Lymphocyte Source (Green).

Networks provide effective models to study complex biological systems, such as gene and protein interaction networks. A weighted gene coexpression network was constructed using adjacency matrix based on superman coefficient. We further studied the topological features to find key nodes in the networks. Genes whose degree was greater than the average degree values were considered as hub genes. Based on the three networks of 7 pathways from All Sources, Blood Source, and Lymphocyte Source, we mapped a Venn diagram.  Figure 4 shows that the intersection of these three sets contained only one gene, PIK3CA. We defined PIK3CA as the common marker of NS. The intersection of All Sources and Blood Source had two genes, namely, PIK3CA and TGFBR2. A total of 4 genes (PIK3CA, CDKN1B, KRAS, and E2F3) existed in All Sources and Lymphocyte Source sets, simultaneously. There were 3 genes that shared in both Blood Sources and Lymphocyte Source: PIK3CA, TRAF6, and CHUK.

### 3.6. Expression Levels of Hub Genes and Common Inflammatory Factors

**Figure 5:**
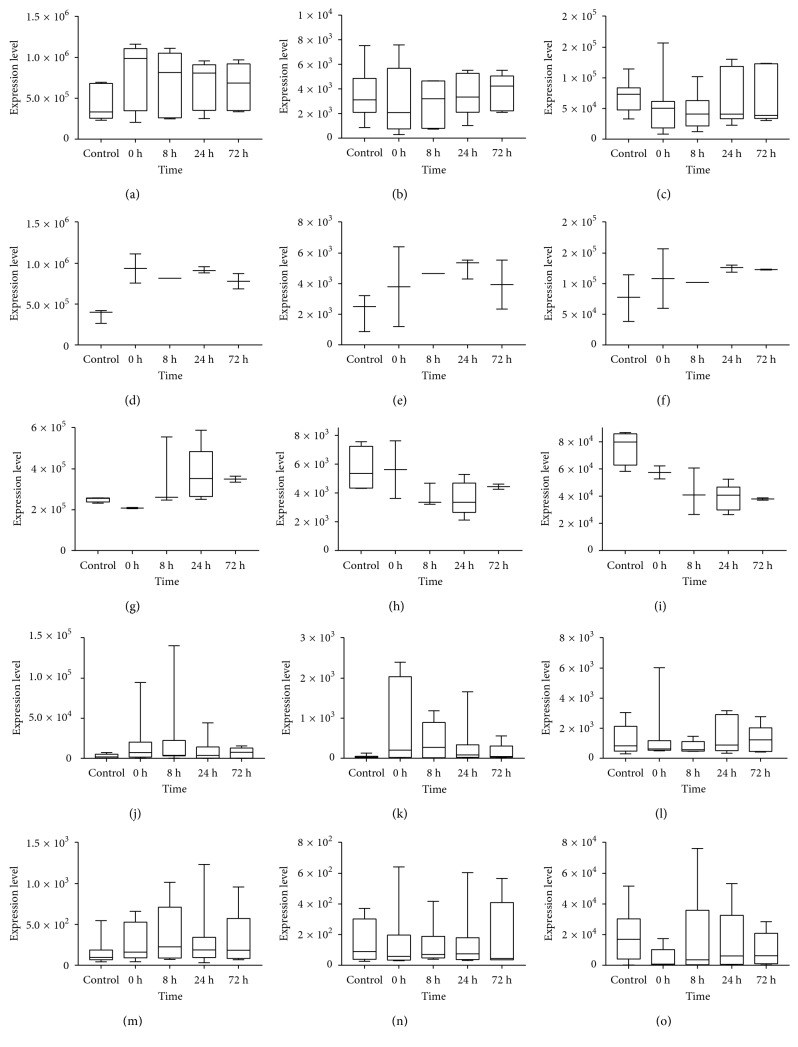
Box-whisker plot of expression levels of genes from GSE11755. (a) GAPDH (internal reference) from All Sources; (b) PIK3CA from All Sources; (c) TGFBR2 from All Sources. (d) GAPDH from Blood Source; (e) PIK3CA from Blood Source; (f) TGFBR2 from Blood Source. (g) GAPDH from Lymphocyte Source; (h) PIK3CA from Lymphocyte Source; (i) TGFBR2 from Lymphocyte Source. Levels of (j) IL-6, (k) IL-10, (l) TNF-*α*, (m) IL-18, (n) IL-7, and (o) IFNA1 from All Sources. *Y*-axis represents the expression levels of genes. *X*-axis represents control and several time points after admission to the paediatric intensive care unit. The box represents the express range and the central line was the median of the data. All graphs were made with GraphPad Prism 7.0.

After analyzing the topological features of networks based on 7 pathways, 7 genes were considered as the hub genes which were described above. In order to investigate the relevance of the hub genes and NS, the expression levels of PIK3CA, TGFBR2, CDKN1B, KRAS, E2F3, TRAF6, and CHUK were further analyzed. As we all know that NS is mainly caused by infections, the levels of proinflammatory genes were also observed, such as tumor necrosis factor alpha (TNF-*α*), interleukin-2 (IL-2), interleukin-6 (IL-6), interleukin-7 (IL-7), interleukin-10 (IL-10), and interferon alpha-1 (IFNA1). Besides, we examined expressions of housekeeping genes GAPDH and beta-catenin (not shown here) aiming at objectively reflecting the changes in hub genes.  Figure 5 shows the expression levels of these genes in Box-whisker plot. We can easily found that PIK3CA levels from common and blood groups of patients after admission to the paediatric intensive care unit had no obvious changes compared with controls, while expression of PIK3CA from Lymphocyte Source significantly decreased. According to the reports, NS is mainly caused by infections; however, there was no significant difference between controls and patients in immune response-related gene expression levels (Figures 5(d)–5(i)). The expressions of CDKN1B, KRAS, E2F3, TRAF6, and CHUK were not displayed here.

## 4. Discussion

In recent years, many new mathematical model methods such as high-dimensional differential equations [25, 26], dynamic Bayesian network [27, 28], and Granger's model [29] were widely used in molecular biology and bioinformatics. According to reports, inchoate changes in gene expression underlying diseases or infections could be calculated by mathematical models. Low et al. [27, 28] used them to analyze the temporal causality between genes on account of changes expressed at many time points. Time-series gene expression experiments are getting more and more popular. This method plays an important role in studying translation and gene regulation. We provide a flexible way to detect common expression patterns in the individual subjects. Elastic-net regression model combined with the MWU test was used in this study. According to this method, both individual gene and gene set changes, which are induced by infection in a subject-specific way, will be detected.

In the classic MWU test, each variable is independent and there is no relationship between them. However, genes are interrelated, in particular within the related signaling pathway. Therefore, we must make some amendments to the classic MWU test, which can be used to accommodate with gene correlation. In our method, we assume that genes in the relative signaling pathway share a common pairwise correlation *q* and the irrelevant genes maintain independence. In the current study based on KEGG enrichment of gene signatures, the results showed that, among several KEGG pathways, the top 3 significant pathways were hsa05220: Chronic myeloid leukemia, hsa04380: Osteoclast differentiation, and hsa05222: Small-cell lung cancer, respectively. Besides, pathways including the proinflammatory cytokine genes were also studied, such as hsa05164: Influenza A, hsa04620: Toll-like receptor signaling pathway, and hsa05168: Herpes simplex infection. Unfortunately, the MWU test showed that there was no difference between the control and common groups in those proinflammatory cytokines included pathways. In order to determine the hub genes, based on the topological characteristics of coexpression networks, PIK3CA was defined as the common marker of NS. Then, TGFBR2, CDKN1B, KRAS, E2F3, TRAF6, and CHUK were also selected as our target molecules.

PIK3CA, an oncogene, encodes the p110 catalytic subunit of class I phosphatidylinositol 3-kinases (PI3Ks), namely, PI3Kp110a. Approximately 4/5 of the mutations in PIK3CA occur in the two hot spots, exon 9 and exon 20. Its mutation not only can reduce the apoptosis of cells but also can promote the infiltration of tumors and increase the activity of its downstream kinase PI3Ks [30]. Under physiological conditions, PIK3CA is expressed in brain, lung, mammary gland, gastrointestinal tract, cervix, and other tissues and has many important physiological functions such as regulation of somatic cell proliferation, differentiation, and survival. PIK3CA is often inactive and usually not easily detected. However, PIK3CA was overexpressed after mutation, which could increase the catalytic activity of PI3Ks and promote cell canceration in tissues. PIK3CA mutation has become the molecular biomarker of many tumors [31–34]. PI3K-Akt-mTOR signaling is associated with the balance between cell proliferation and survival and plays a major role not only in tumor growth but also in the potential response of cancer treatment, such as wortmannin and LY294002 [35, 36]. Unfortunately, it seems that there is no direct correlation between PIK3CA and NS in the existing literature.

TGFBR2, transforming growth factor, beta receptor II, is a tumor suppressor gene. The encoded protein is a transmembrane protein that has a protein kinase domain, forms a heterodimeric complex with another receptor protein, and binds TGF-beta. Heterozygous mutations in TGFBR2 play an important role in Marfan syndrome, which is an extracellular matrix disorder with cardinal manifestations in the eye, skeleton, and cardiovascular systems [37]. Several recent reports showed that inducible ablation of TGF-*β* receptor type 2 signaling was able to limit hepatic stellate cells and fibrosis and attenuates tumor-associated inflammation [38]. TGF-*β* acts as a key regulator of immune cells, epithelium, in inflammatory bowel disease [39]. Many studies have shown that TGFBR2 signaling was associated with inflammatory-related diseases. But, whether TGFBR2 and NS are related is still a mystery. CDKN1B, a cyclin-dependent kinase inhibitor 1B, can bind to and prevent the activation of cyclin E-CDK2 or cyclin D-CDK4 complexes and thus controls the cell cycle progression at G1. KRAS is a gene that acts as an on/off switch in cell signaling and controls cell proliferation. Most of the target molecules we selected were related to the cell proliferation and tumorigenesis. While very few literature studies report the correlation between them and NS. This study gave us a new enlightenment for neonatal sepsis research. However, there were some limitations to our study. Firstly, PIK3CA and other molecular biomarkers were predictive biomarkers of *n*, and further experimental verification should be conducted to verify our results. Besides, whether the workflow was suitable for other analysis or another database is a question.

## 5. Conclusion

In conclusion, a comprehensive process of data in datasets of NS was conducted in our research. Then, the function and signaling pathways of NS were presented systematically by the cutting edge models. Finally, based on the potential pathways and their topological characteristics of coexpression networks, several critical genes for NS were identified. PIK3CA was defined as the common marker of NS. However, the current study was based on the previous reports and more clinical evidence results were needed.

## Figures and Tables

**Table 1:** Top 6 differentially expressed pathways according to the KEGG analysis.

Pathway_name	*p* value	FDR	Gene count
Hsa04740: Olfactory transduction	1.25*E* − 138	3.59*E* − 136	59
Hsa05206: MicroRNAs in cancer	1.07*E* − 23	1.53*E* − 21	133
Hsa04080: Neuroactive ligand-receptor interaction	2.84*E* − 10	2.03*E* − 08	179
Hsa04110: Cell cycle	2.42*E* − 10	2.03*E* − 08	117
Hsa04380: Osteoclast differentiation	1.45*E* − 09	8.27*E* − 08	122
Hsa00830: Retinol metabolism	2.47*E* − 09	1.0*E* − 07	23

**Table 2:** *p* values of MWU test of sample groups.

Groups	KEGG pathways	*p* values
All Sources	hsa05220: Chronic myeloid leukemia	0.0457024

Blood Source	hsa05120: Epithelial cell signaling in *Helicobacter pylori* infection	0.0357933
	hsa04380: Osteoclast differentiation	0.0380088
	hsa04666: Fc gamma R-mediated phagocytosis	0.0415344

Lymphocyte Source	hsa05222: Small-cell lung cancer	0.0150380
	hsa04660: T cell receptor signaling pathway	0.0412070
	hsa05219: Bladder cancer	0.0463346

Monocyte Source	None	

hsa: *Homo sapiens* (human); *p* values < 0.05 significant difference.

## Data Availability

The data used to support the findings of this study are available from the corresponding author upon request.
